# Quercetin improves contrast-induced acute kidney injury through the HIF-1α/lncRNA NEAT1/HMGB1 pathway

**DOI:** 10.1080/13880209.2022.2058558

**Published:** 2022-05-19

**Authors:** Min Luo, Ziyu Liu, Zongren Hu, Qinghu He

**Affiliations:** a Department of Nephrology, The Second Xiangya Hospital, Central South University, Changsha, Hunan Province, China; b Department of Rehabilitation Medicine and Health Care, Hunan University of Medicine, Huaihua, Hunan Province, China; c College of Traditional Chinese Medicine, Hunan University of Chinese Medicine, Changsha, Hunan Province, China; d College of Integrated Traditional Chinese and Western Medicine, Hunan University of Chinese Medicine, Changsha, Hunan Province, China

**Keywords:** CI-AKI cell model, HuangKui, cell injury and apoptosis, NEAT1

## Abstract

**Context:**

The risk of contrast-induced acute kidney injury (CI-AKI) is increasing and the harm is great. Quercetin is the main active component in *Abelmoschus manihot* (L.) Medik (Malvaceae) and was reported to reduce the expression of HIF-1α.

**Objective:**

We investigate whether quercetin improves the CI-AKI through the HIF-1α/lncRNA NEAT1/HMGB1 pathway.

**Materials and methods:**

HK-2 cells were treated with iohexol (200 mg/mL) for 6 h to establish a CI-AKI model. Quercetin (20 μM) was administered to CI-AKI cells cultured in dishes for 24 h. Cell morphology was observed by a fluorescence microscope. MTT and TUNEL assays were used to detect cell survival rate and apoptosis. Relative mRNA levels were measured by qRT-PCR. Protein levels were detected using western blotting. IL-6 and TNF-α protein levels were tested by Elisa assay. Targeting binding sites of HIF-1α and lncRNA NEAT1 were detected by luciferase assay.

**Results:**

The IC_50_ value of quercetin was 163.25 μM. The expression levels of HIF-1α, lncRNA NEAT1 and HMGB1 were upregulated in the CI-AKI cell model. Quercetin diminished cell injury and apoptosis via inhibiting HIF-1α. Silencing of HIF-1α targeting lncRNA MEAT1 diminished cell injury and apoptosis. Silencing lncRNA NEAT1 has the same effect via suppressing HMGB1 expression. Collectively, quercetin diminished cell injury and apoptosis in CI-AKI cell model via the inhibition of HIF-1α on lncRNA NEAT1/HMGB1 signalling pathway.

**Discussion and conclusions:**

Quercetin diminished cell injury and apoptosis in CI-AKI cell mode via the inhibition of HIF-1α on the lncRNA NEAT1/HMGB1 signalling pathway, offering a potential novel therapeutic target for CI-AKI therapy.

## Introduction

Contrast is widely used in clinical practice, such as enhanced CT and cardiac catheterisation (Morcos et al. 2019). The incidence of contrast-induced acute kidney injury (CI-AKI) increases year by year, with an incidence ranging from 3 to 25% depending on risk factors, while for high-risk groups, the incidence is up to 30–50% (Cantais et al. 2016). Contrast-induced acute kidney injury (CI-AKI) is characterised by a decline in kidney function within the first 48–72 h following contrast administration, in the absence of alternative aetiologies (Mehran and Nikolsky 2006; McCullough et al. 2016). CI-AKI was reported to be the third major cause of AKI in hospitalised patients (Gleeson and Bulugahapitiya 2004; Do 2017). After the occurrence of CI-AKI, the incidence of long-term renal failure is increased. Even worse, CI-AKI is a rather detrimental complication that closely correlates with high morbidity and mortality (Liang et al. 2021). Percutaneous coronary intervention (PCI) in patients with atrial fibrillation (AF) has changed in recent years with new data from large randomised trials and updates to clinical guidelines (Kwon et al. 2021). Therefore, the number of coronary intervention cases in China will continue to increase, and the risk of CI-AKI is still increasing. Despite the terrible harmfulness of CI-AKI, there is still no best clinical practice to prevent it. Thus, it is very necessary to comprehensively understanding the pathological mechanism of CI-AKI and further developing effective therapeutical strategies.

*Abelmoschus manihot* (L.) Medik (Malvaceae), also called “Huang Kui” in Chinese, is an annual flowering herb plant. The flower of *Abelmoschus manihot* has various obvious biological activities, such as anti-inflammatory, analgesic, antioxidation, anticoagulation, anti-myocardial ischaemia and necrosis, protection against ischaemic brain injury, delay renal tubular damage and fibrosis, reduce blood sugar, promote angiogenesis, etc. Flavonoids, including quercetin, are the main active components in *Abelmoschus manihot* (Li et al. 2021). There is growing evidence suggesting that quercetin has therapeutic potential for the prevention and treatment of different diseases, including cardiovascular disease, cancer, and neurodegenerative disease. Mechanistically, quercetin has therapeutic potential for the prevention and treatment of neurodegenerative diseases such as Alzheimer’s disease (AD) and PD because of its antioxidant and anti-inflammatory properties and its ability to cross the blood-brain barrier (Ansari et al. 2009; Haleagrahara et al. 2011; Ishisaka et al. 2011). Previous reports suggested that quercetin significantly attenuated aneurysm growth via downregulating hypoxia-inducible factors 1α (HIF-1α) in a mouse model of abdominal aortic aneurysm (Wang et al. 2020). Additionally, a study has indicated that quercetin suppressed radioresistance by inhibiting the expression of HIF-1α (Li and Wei 2018). In addition, according to a previous study that HIF-1α, lncRNA nuclear enriched abundant transcript 1 (NEAT1) can be indicated as a biomarker in the process of CI-AKI (Efremova et al. 2019; Lin et al. 2021). The lncRNA NEAT1 promoted sepsis-inflammatory responses and acute kidney injury (AKI) (Wang and Guo 2020). Different investigations have shown that inhibition of lncRNA NEAT1 can prevent acute injury and inflammatory response of alveolar epithelial cells induced by LPS through the High Mobility Group Box-1 (HMGB1)/RAGE signalling pathway (Zhou et al. 2020). In addition, some studies reported that HMGB1 plays an important role in a variety of live injuries, such as non-alcoholic fatty liver disease (NAFLD), damage-associated lethal hepatitis (Wang et al. 2012; Lin et al. 2020). In the case of acute liver injury, it leads to aseptic inflammation and other reactions and also regulates specific cell death responses in chronic liver injury (Fang et al. 2020). Furthermore, there is also a report of HMGB1-targeting therapies in acute liver failure (ALF) models (Yamamoto and Tajima 2017). Kidney Injury Molecule-1 (KIM-1) is a type 1 transmembrane protein with immunoglobulin and mucin domains that are highly expressed in the proximal tubule of post-ischaemic rat kidneys (Han et al. 2002). NGAL (neutrophil gelatinase-associated lipocalin) is a lipocalin superfamily protein with a size of 25 kDa. Although it was first discovered in active neutrophils, NGAL can be produced by a variety of cells, including kidney tubular cells, in response to diverse stressors (Soni et al. 2010).

Even though quercetin plays an important role in improving various diseases, there are no data available that whether quercetin improves the process of CI-AKI, and if so, what are the underlying mechanisms. Here, we investigate whether quercetin improves the pathological process of CI-AKI using a human renal tubular epithelial cell (HK-2) model and whether the impact of quercetin on CI-AKI is mediated by HIF-1α/lncRNA NEAT1/HMGB1 signalling pathway.

## Materials and methods

### Recovery and subculture of HK-2 cells

The human proximal tubule epithelial cell line HK-2 was purchased from Stem Cell Bank (Chinese Academy of Sciences, Shanghai, China). The culture medium (DMEM/F12; Gibco; Thermo Fisher Scientific, Inc., Waltham, MA, USA) containing 10% foetal bovine serum (FBS; Gibco; Thermo Fisher Scientific, Inc., Waltham, MA, USA) and antibiotics (100 IU/mL penicillin and 100 μg/mL streptomycin) was used for HK-2 cells culture. Cells were cultured at 37 °C in a humidified atmosphere condition containing 95% O_2_ and 5% CO_2_. Cells were subcultured when cells reached 80% confluence.

### Establishment of CI-AKI cell model

HK-2 cells were incubated in the DMEM/F12 medium for up to 24 h. HK-2 cells were divided into five groups, which were treated with iohexol (200 mg/mL) for 0, 2, 6, 12, 24 h, respectively. The alternation of cell morphology was observed using phase-contrast microscope.

### Evaluation of cell viability by MTT

Cell viability was assessed using previous methods (Sun et al. 2016). In brief, cells cultured in 96-well plates were incubated with DMEM supplemented with MTT [3-(4,5-dimeth-ylthiazol-2-yl)-2,5-diphenyltetrazolium bromide] solution (5 mg/mL, Sigma) for 4 h at 37 °C after washing with 0.1 M PBS. To dissolve the crystal formazan, a 200 μL dimethyl sulfoxide solution was added to each well. At last, absorbance was captured at 490 nm using a microplate reader (Bio-Rad, Hercules, CA, USA) to evaluate the cell viability. Pharm statistical package (Springer Verlag, New York, NY) was used to evaluate the half-maximal inhibitory concentration (IC_50_) values.

### TUNEL assay

The terminal deoxynucleotidyl transferase-mediated dUDP nick-end labeling (TUNEL) technique was performed to assess the cell apoptosis. We make use of a kit purchased from Roche company. HK-2 cells were cultured on sterile glass coverslips in a 6-well plate and stimulated with iohexol for 0, 2, 6, 12 and 24 h. The apoptosis of HK-2 cells was assessed by the TUNEL assay following the manufacturer’s recommendations. TUNEL-positive cells were counted under the light microscope. The rate of apoptosis in HK-2 cells was calculated by dividing the number of TUNEL positive cells to a population of 100 counted cells per microscopic field (magnification ×40 or ×100).

### Luciferase reporter assay

The 3′ untranslated region of the wild-type (WT) or mutant lncRNA-NEAT1 (or UCK2) sequence was inserted into the psiCHECK-2 luciferase reporter vector (Promega Corporation, Madison, WI, USA). Cells were lysed, then luciferase activity was assessed by the Dual-Luciferase Reporter Assay System (Promega Corporation) after 48 h co-transfection with the luciferase reporter vector and HIF-1α (or AllStars Negative Control). Renilla luciferase activity was normalised to firefly luciferase activity. HK-2 cells or HIF-1α knock-down cells were transfected with luciferase reporter vectors containing the WT or mutant putative hypoxia response element (HRE) sequence (ACGTGC) and then treated with CoCl_2_ for 24 h. A similar luciferase reporter assay was performed to assess the effect of HIF-1α on the promoter of lncRNA-NEAT1.

### Western blotting

Cultured HK-2 cells were harvested for western blotting experiments. Briefly, we used an ice-cold RIPA buffer to extract the total protein that was quantified using a BCA Protein Detection Kit (Pierce, Rockford, IL, USA). Then, equal amounts of protein were subjected to 12% SDS-PAGE, following transfer to nitrocellulose membranes. Next, the membranes were blocked in TBST buffer containing 5% BSA for 1 h at room temperature and incubated overnight at 4 °C with primary antibodies against HMGB1 (Abcam, 1:1000, ab18256), HIF-1α (Santa Cruz, 1:1000, sc13515), Kim-1 (Thermo Fisher, 1:1000, PA5-79345), NAGL (Abcam, 1:2000, ab63929), IL-6 (Santa Cruz, 1:1000, sc-130326), TNF-α (Santa Cruza, 1:1000, sc133192). Then the membrane was incubated with horseradish peroxidase-conjugated secondary antibodies, and treated with ECL Reagent (Beyotime) to visualise the immunoreactive bands. The bands were quantified using a Gel Doc™ XR imaging system (Bio-Rad Laboratories, Hercules, CA, USA) and Image J software. For normalisation, β-actin was employed as an internal standard.

### Quantitative reverse transcription polymerase chain reaction (qRT-PCR) analysis

HK-2 cells were cultured for up to 48 h, and then total RNA from cells was prepared and synthesised into cDNA. Then, qRT-PCR was performed to detect expression levels of lncRNA NEAT1, HMGB1 and HIF-1α using a SYBR® Premix Ex Taq™ II Kit (Takara, Otsu, Japan). The HIF-1α-specific primers were designed as previously described (Wang et al. 2020) and obtained by Shanghai Sheng Gong Biological Company (Shanghai, China). Primers for the other genes were as follows: lncRNA NEAT1, forward 5′-TGTCCCTCGGCTATGTCAGA-3′, reverse 5′-GAGGGGACGTGTTTCCTGAG-3′; HMGB1 forward 5′-TGGACTGCTCAGGAAAC-3′, reverse 5′-AGGGGCAAACCGTAAT-3′. β-Actin was used as an internal reference to quantify the expression of target genes. All samples were analysed using an Applied Biosystems 7300 Real-Time PCR System (Applied Biosystems, Foster City, CA, USA). The 2-ΔΔCt equation was used to calculate the results.

### ELISA assay

Cytokine concentration in HK-2 cells was determined by ELISA single kits for IL-6 (EH2IL6), TNF-a (EH3TNFA), according to the manufacturer’s instructions (Invitrogen).

### Statistical analysis

One-way ANOVA followed by a Bonferroni comparison test and Student’s *t*-test (two-tailed) were used to determine statistical significance with a *p-*value threshold set at <0.05.

## Results

### The expression levels of HIF-1α, lncRNA NEAT1 and HMGB1 were upregulated in the CI-AKI cell model

**Figure 1.**
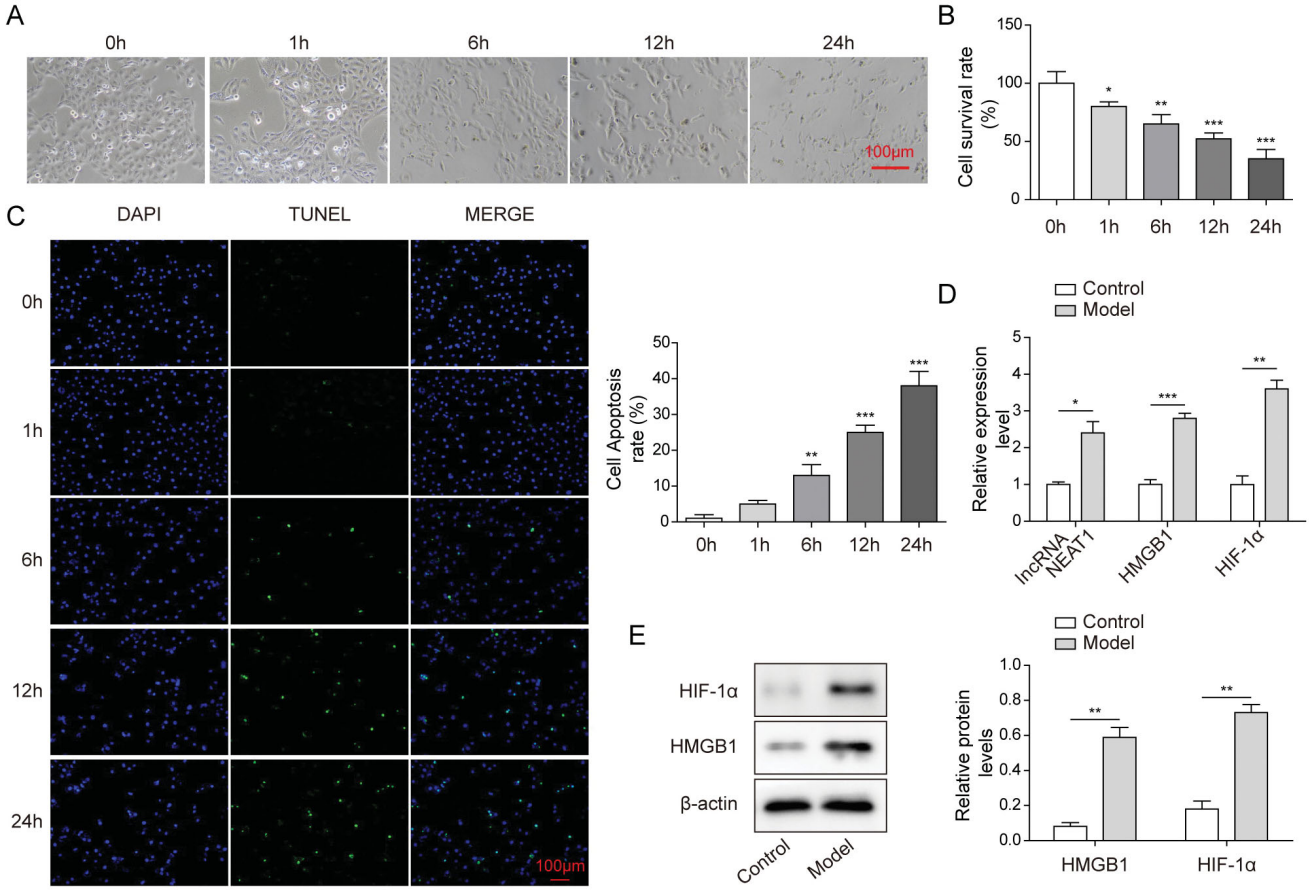
The expression levels of HIF-1α, lncRNA NEAT1 and HMGB1 were upregulated in the CI-AKI cell model. (A) HK-2 cells were treated with iohexol (200 mg/mL) for 0, 1, 6, 12 and 24 h. (B) Cell survival rate was assessed by MTT assay. Cell survival rate was gradually decreased with culture time increasing. (C) Cell apoptosis was detected using a TUNEL assay. (D) Expression levels of HIF-1α, lncRNA NEAT1 and HMGB1 were detected by the qRT-PCR assay. The bar chart shows that expression levels of HIF-1α, lncRNA NEAT1 and HMGB1 were increased in the CI-AKI group compared with the control group. (E) Western blotting was performed to detect the protein level of HIF-1α and HMGB1. The data were expressed as mean ± SD. *p < 0.05, **p < 0.01.

The IC_50_ values of quercetin was 163.25 μM. Then, CI-AKI cell model was established for further study. HK-2 cells were cultured for up to 24 h, and cells were divided into five groups based on their culture time, namely 0, 1, 6, 12, and 24 h groups. Iohexol (200 mg/mL) was added to the five cell groups to simulate the damage of contrast on the kidney. Cell morphology was observed making use of the phase-contrast microscope, and we found that HK-2 cells in the 0 h group expressed a normal adherent growth, showing elliptical epithelial-like cell morphology (Figure 1(A)). Next, MTT was used to detect the cell activity, respectively. The results indicated that compared with the control group (0 h), the cell activity decreased strikingly (Figure 1B), and the apoptosis rate was markedly increased with the increase of CI-AKI model establishment time (Figure 1C). The above results showed that with the increase in model establishment time, the survival rate of cells decreased, and the apoptosis rate increased significantly. According to the above research and the experiment of this study, the model establishment time was selected as 6 h. Next, we cultured HK-2 cells for 6 h with (model) and without (control) iohexol addition. We first detected whether the mRNA levels of lncRNA NEAT1, HMGB1, and HIF-1α were altered in the CI-AKI model using the qPCR technique. Our results revealed that the mRNA levels were strikingly increased in model groups (Figure 1D). Based on these findings, we speculated that the protein level of HIF-1α and HMGB1 will be also increased. As speculated, the expression levels of HIF-1α and HMGB1 were markedly increased in the model group (Figure 1E). Taken together, these findings suggested that the expression levels of HIF-1α, lncRNA NEAT1, and HMGB1 were significantly upregulated in the CI-AKI cell model.

### Quercetin reduced cell injury and apoptosis in CI-AKI model via inhibiting HIF-1α

**Figure 2.**
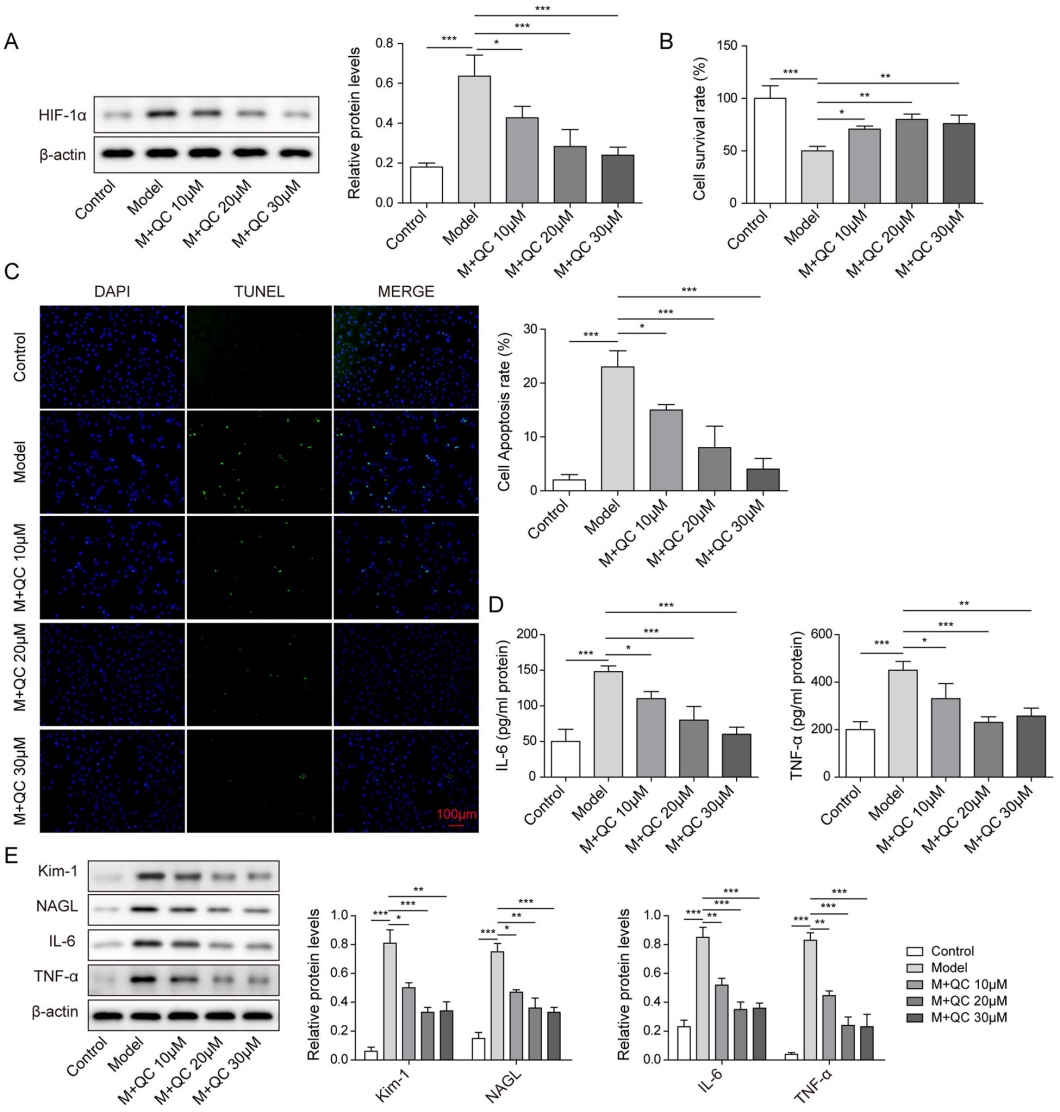
Quercetin diminished cell injury and apoptosis in the CI-AKI model via inhibiting the expression of HIF-1α. (A) HIF-1α was assessed by western blotting in different groups treated with different concentrations of quercetin. (B) Cell survival rate was gradually increased in groups treated with 10, 20, and 30 μM quercetin. (C) Cell apoptosis rate was gradually decreased in model groups with quercetin concentration increased. (D) The expression levels of IL-6 and TNF-α were reduced with quercetin concentration increased in the CI-AKI model. (E) The expression levels of Kim-1, NAGL, IL-6 and TNF-α were decreased with the concentration of quercetin increased. The data were expressed as mean ± SD. *p < 0.05, **p < 0.01.

A previous study showed that HIF-1α is a biomarker for CI-AKI. We next investigated whether quercetin inhibits the expression of HIF-1α, and if so, which is the best concentration for the next experiments. We divided HK-2 cells into five groups, that were control group, model group, model + 10 μM quercetin group (M + QC 10 μM), model + 20 μM quercetin group (M + QC 20 μM), model + 30 μM quercetin group (M + QC 30 μM). The expression of HIF-1α was assessed using western blot analysis. Our results showed that all three concentrations of quercetin significantly reduced the expression of HIF-1α in the CI-AKI model, but 20 and 30 μM quercetin had a better effect than 10 μM quercetin, but there was no difference between these two groups (Figure 2A).

We next explored the effect of different concentrations of quercetin on HK-2 cell survival rate. In Figure 2B, the result showed that the cell survival rate was strikingly increased in all quercetin groups compared to the model group. Additionally, we found that the apoptosis rates of HK-2 cells treated with three concentrations of quercetin were significantly decreased compared to the model group, and the apoptosis was less than 10% in a group of 20 μM quercetin (Figure 2C). Tissue necrosis factor-alpha (TNF-α) and interleukin-6 (IL-6) are pro-inflammatory cytokines that contribute to the improvement of inflammatory and decreasing its expression will release the inflammatory (Oza et al. 2021). Next, we sought to explore if quercetin reduces the expression levels of IL-6 and TNF-α. Quercetin with concentrations of 10, 20, 30 μM was applied to the cell of the CI-AKI model. Elisa assay was used to detect the expression levels of IL-6 and TNF-α. Results indicated that all three concentrations of quercetin significantly reduced the expression level of IL-6 and TNF-α in the CI-AKI model. Moreover, there was no difference in effect between quercetin of 20 and 30 μM (Figure 2D).

Previous studies reported that kidney injury molecule 1 (Kim-1) and neutrophil gelatinase-associated lipocalin (NGAL) were biomarkers of chronic renal failure, and the expression level of these two factors was markedly increased in the model of renal failure (Li et al. 2020). So, we sought to detect the expression levels of Kim-1, NGAL, IL-6 and TNF-α using western blotting. Our results showed that the expression levels of Kim-1, NGAL, IL-6 and TNF-α were significantly increased in the model group compared to the control group. After 10, 20 and 30 μM quercetin were applied to the cells in the model groups, the expression levels of Kim-1, NGAL, IL-6 and TNF-α were sharply decreased with no difference between 20 and 30 μM quercetin groups. These results indicated that quercetin strikingly alleviated the cell injury and apoptosis in the CI-AKI model via attenuating the expression of HIF-1α with the best effect at a dose of 20 μM.

### Silencing HIF-1α inhibit damage and apoptosis in CI-AKI cells via targeting lncRNA NEAT1

**Figure 3.**
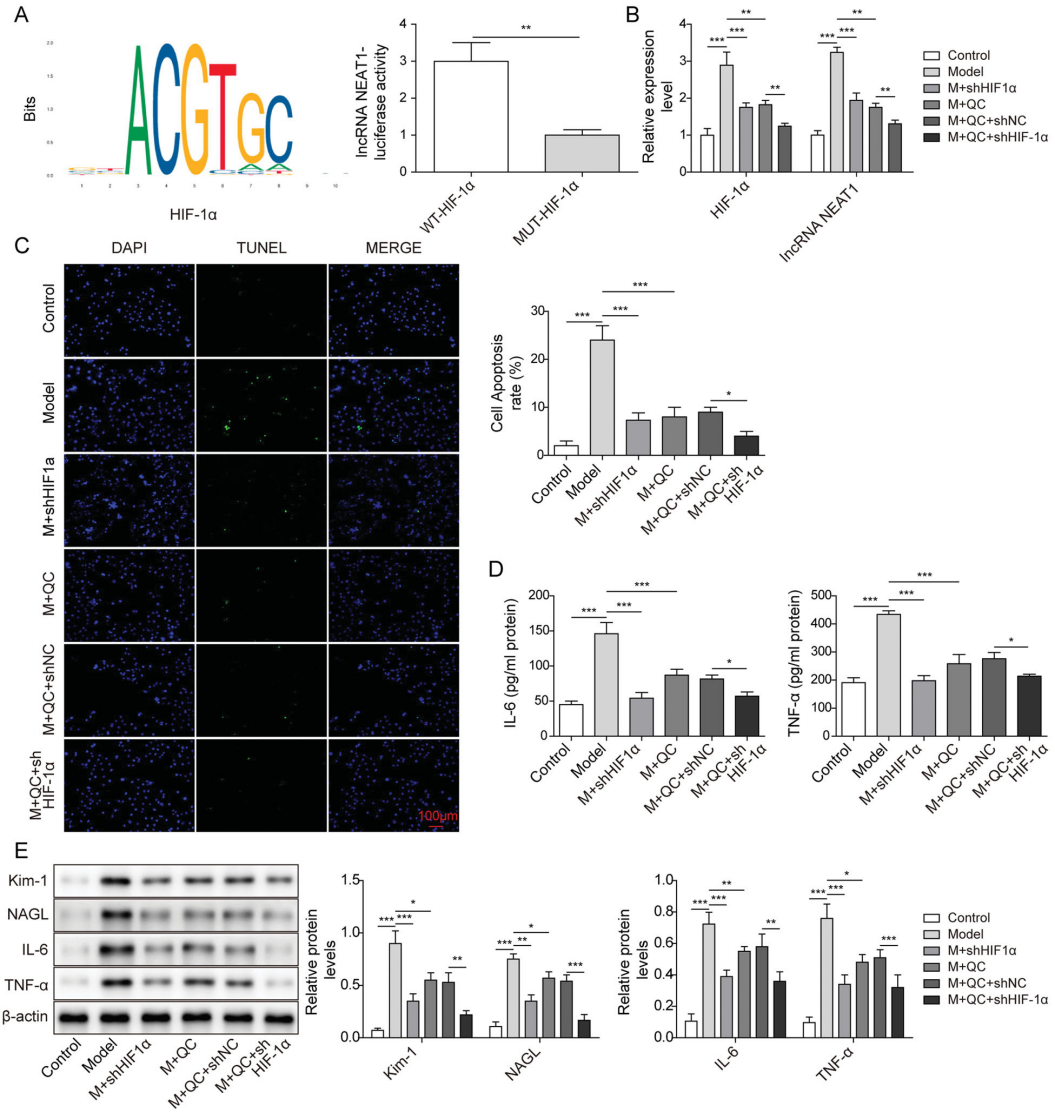
Silencing HIF-1α inhibited cell injury and apoptosis in the CI-AKI model via targeting lncRNA NEAT1. (A) The putative binding sites of HIF-1α and lncRNA NEAT1. A luciferase reporter assay was performed to validate the interplay between HIF-1α and lncRNA NEAT1. The colored letters represent the Motif logo, and each column of the motif logo represents each location. The larger the base, the more likely it is to appear at that location. (B) The relative mRNA expression levels of HIF-1α, TNF-α in various groups were examined by qRT-PCR. (C) Cell apoptosis rate was detected in different groups by TUNEL assay. (D) The protein expression levels of IL-6 and TNF-α were tested by Elisa assay. (E) Expression levels of Kim-1, NAGL, IL-6 and TNF-α were detected between the Model and Ctrl group, M + QC group and Model group, M + QC + sh-HIF-1α and M + QC group. The data were expressed as mean ± SD. *p < 0.05, **p < 0.01.

Previous studies showed that lncRNA NEAT1 promoted inflammatory responses and acute kidney injury (Wang and Guo 2020). We assumed that silencing HIF-1α directly inhibited cell injury and apoptosis via targeting lncRNA NEAT1. We first tested whether there existed target binding sites between HIF-1α and lncRNA NEAT1 using bioinformatics methods. As shown in Figure 3A, there existed a target binding sites sequence between HIF-1α and lncRNA NEAT1. Luciferase activity of lncRNA NEAT1 was markedly increased in the WT-HIF-1α group compared with that in the HIF-1α group, indicating close binding between HIF-1α and lncRNA NEAT1 (Figure 3A). Next, we divided cells into six groups, including the control group (control), model group (M), M + sh-HIF-1α, M + QC (quercetin), M + QC + sh-NC, M + QC + sh-HIF-1α group, to go on our experiments. We first tested the mRNA expression levels of HIF-1α and lncRNA NEAT1 in the six groups using qRT-PCR. We found that the expression of HIF-1α and lncRNA NEAT1 in the Model group was significantly higher than that in the control group, which was greatly reduced by sh HIF-1α. But, the expression of HIF-1α and lncRNA NEAT1 in the M group were greatly reduced in the M + QC group, which was consistent with our previous results. We employed sh-HIF-1α to disturb the expression of HIF-1α and found that the expression of HIF-1α and lncRNA NEAT1 in the M + QC + sh-HIF-1α group was strikingly reduced compared to M + QC + sh-NC group (Figure 3B). We further tested the cell apoptosis, and our results showed that the cell apoptosis rate of the Model group was significantly increased compared to the control group, but in the M + sh HIF-1α and M + QC group, cell apoptosis was significantly decreased. In the M + QC + sh-HIF-1α group, cell apoptosis was also strikingly reduced compared to the M + QC group (Figure 3C). IL-6 and TNF-α are two inflammatory factors, and decreasing their expression will relief the inflammatory response. The expression of IL-6 and TNF-α were sharply increased in the Model group, but significantly decreased in M + sh HIF-1α and M + QC group which is similar to the results in Figure 2D and 3D). In addition, we found that the expression was also reduced in the M + QC + sh-HIF-1α group in which HIF-1α was disturbed compared with the M + QC + sh-NC group (Figure 3D). Next, the expression levels of Kim-1, NAGL, IL-6 and TNF-α were assessed by western blotting. Compared to the control group, the expression levels of Kim-1, NAGL, IL-6 and TNF-α were sharply increased, however, after the application of quercetin to the Model group, the expression of these factors was strikingly decreased (Figure 3E). Moreover, after disturbing the expression of HIF-1α, the expression levels of Kim-1, NAGL, IL-6 and TNF-α were sharply reduced (Figure 3E). Taken together, these findings suggested that silencing HIF-1α inhibits cell injury and apoptosis in the CI-AKI model via targeted inhibition of lncRNA NEAT1.

### Silencing lncRNA NEAT1 inhibit injury and apoptosis of CI-AKI cells by attenuating the expression of HMGB1

**Figure 4.**
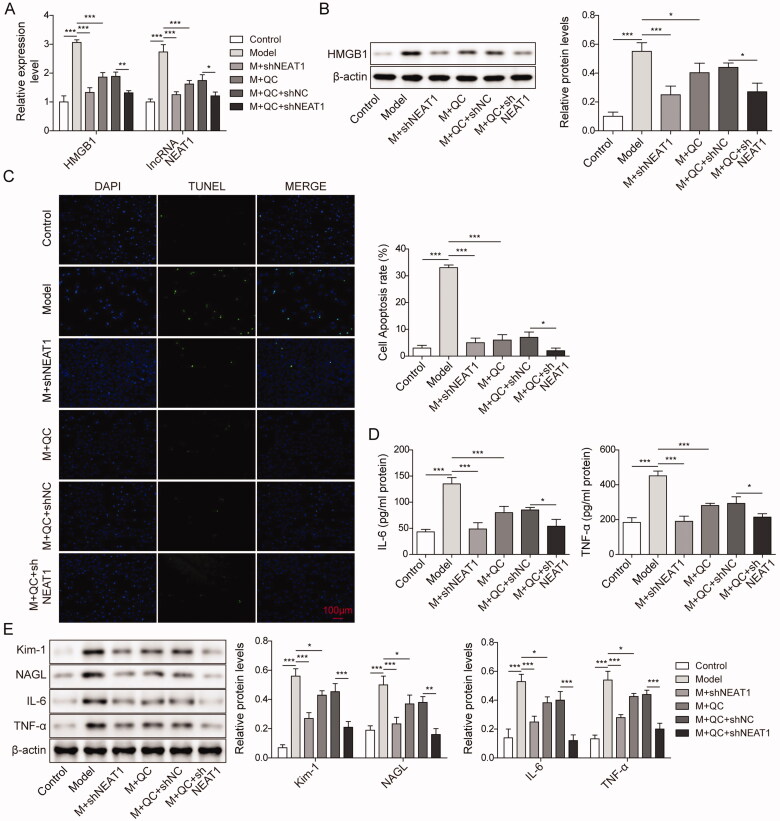
Silencing of lncRNA NEAT1 improved cell injury and apoptosis in the CI-AKI model by inhibiting HMGB1. (A, B) Relative mRNA expression of lncRNA NEAT1, HMGB1 in cells of CI-AKI model, and cells in M + QC + sh-NEAT1 and M + QC + sh-NC group using qRT-PCR. Protein expression of HMGB1 was assessed by western blotting. HK-2 cells were subjected for the analysis of (C) cell apoptosis rate, (D) expression of IL-6 and TNF-α, (E) protein levels of Kim-1, NAGL, IL-6, TNF-α between M + QC + sh-NEAT1 and M + QC + sh-NC group. The data were expressed as mean ± SD. *p < 0.05, **p < 0.01.

We speculated that HMGB1 was the downstream signal factor of lncRNA NEAT1. To address this, we performed sh-NEAT1 to silence the expression of NEAT1. HK-2 cells were divided into six groups, which were including the control group (control), Model group (M), M + sh-NEAT1, M + QC (quercetin) group, M + QC + sh-NC (sh normal control) group, M + QC + sh-NEAT1 group. The mRNA levels of lncRNA NEAT1 and HMGB1 were assessed by qRT-PCR, and protein expression of HMGB1 was detected using Western blotting. As shown in Figure 4A, B, the expression of lncRNA NEAT1 and HMGB1 were the highest in the Model group, and the application of sh-NEAT1 in M + sh NEAT1 and M + QC + sh-NEAT1 groups strikingly attenuated the lncRNA NEAT1 and HMGB1 expression compared with M + QC + sh-NC group, suggesting that sh-NEAT1 attenuate NEAT1 level and HMGB1 is downstream of NEAT1. As to the rate of cell apoptosis, it is the highest in the Model group, but in M + sh NEAT1 and M + QC + sh-NEAT1 groups, the rate was sharply reduced compared with the M + QC + sh-NC group (Figure 4C). We next tested the expression levels of IL-6 and TNF-α by Elisa assay. Our results indicated that the expression levels of IL-6 and TNF-α were the highest among all the groups. Moreover, it was greatly decreased in the M + QC + shNEAT1 group compared to the M + QC + sh-NC group (Figure 4D). Additionally, we found that the protein expression level of Kim-1, NAGL, IL-6 and TNF-α was the highest in the Model group and upon application of sh-NEAT1, the expression levels of these factors were significantly attenuated in M + sh NEAT1 and M + QC + shNEAT1 groups compared with M and M + QC + sh-NC group, respectively (Figure 4E). These results indicated that silencing lncRNA NEAT1 inhibited the expression of HMGB1 and further relief cell injury and apoptosis in the CI-AKI model.

### Quercetin inhibits the lncRNA NEAT1/HMGB1 pathway through HIF-1α and further relieves the injury and apoptosis of CI-AKI cells

**Figure 5.**
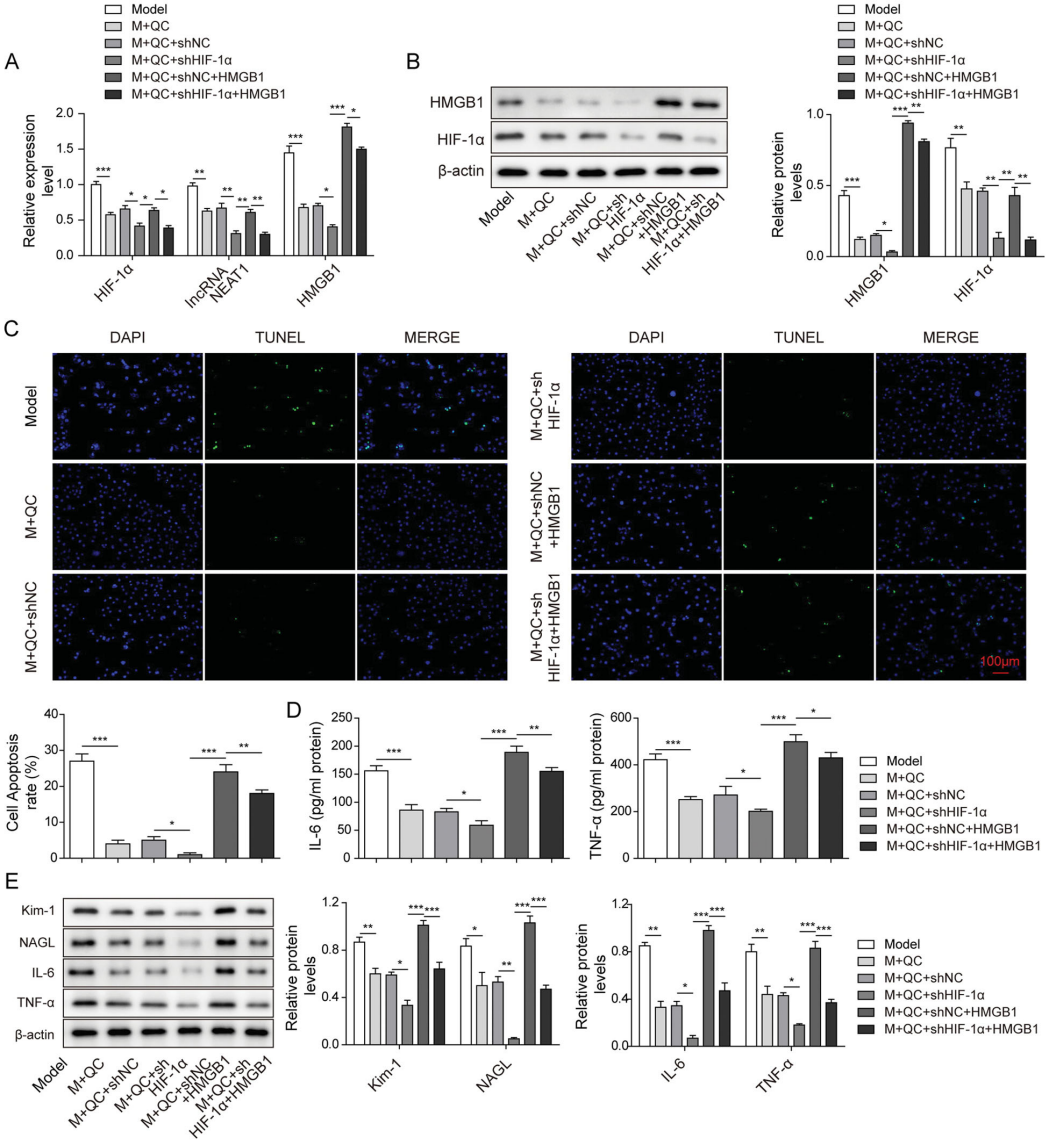
Quercetin diminished cell injury and apoptosis in the CI-AKI model via inhibition of HIF-1α on the lncRNA NEAT1/HMGB1 signalling pathway. (A, B) Relative mRNA and protein expression level of HIF-1, lncRNA NEAT1 and HMGB1 in HK-2 cells of Model, M + QC, M + QC + sh NC, M + QC + sh HIF-1α, M + QC + sh NC + HMGB1, and M + QC + sh HIF-1α + HMGB1 group was measured by qRT-PCR and western blotting. HK-2 cells were subjected for the analysis of (C) cell apoptosis rate, (D) Elisa levels of IL-6 and TNF-α, (E) protein expression of IL-6, TNF-α, Kim-1, NAGL, IL-6 and TNF-α between M + QC and M + QC + sh-NC, M + QC + sh-NC and M + QC + sh-HIF-1, M + QC + sh-NC + HMGB1 and M + QC + sh-HIF-1α+ HMGB1 group. The data were expressed as mean ± SD. *p < 0.05, **p < 0.01.

Based on our previous conclusions that quercetin inhibited the expression of HIF-α; inhibiting HIF-α decreased the expression of lncRNA NEAT1 and inhibition of lncRNA NEAT1 also reduced the expression of HMGB1, we next aimed to explore whether quercetin inhibits the lncRNA NEAT1/HMGB1 pathway through HIF-1α and further relieves the injury and apoptosis of CI-AKI cells. HK-2 cells were divided into six groups, the were Model (M) group, M + QC group, M + QC + sh-NC group, M + QC + sh-HIF-1α group, M + QC + sh-NC + HMGB1 group, M + QC + sh-HIF-1α + HMGB1 group. We found that the expression levels of lncRNA NEAT1, HMGB1 and HIF-1α in the M + QC + sh-NC group were similar to that in the M + QC group. After attenuating the expression of HIF-1α in the M + QC + sh-HIF-1α group, the expression levels of lncRNA NEAT1, HMGB1 and HIF-1α were strikingly downregulated (Figure 5A, B). Moreover, the expression of HIF-1α, lncRNA NEAT1and HMGB1 was significantly attenuated in the M + QC + sh-HIF-1α + HMGB1 group compared to the M + QC + sh-NC + HMGB1 group (Figure 5A, B). Additionally, we found that the cell apoptosis rate of the M + QC + sh-NC group had no significant difference compared with the M + QC group (Figure 5C). But in the M + QC + sh-HIF-1α group, the cell apoptosis rate was significantly attenuated compared to the M + QC + sh-NC group which was consistent with our above results (Figure 5C). Moreover, the cell apoptosis rate in the M + QC + sh-HIF-1α + HMGB1 group was also reduced when compared with the M + QC + sh-NC + HMGB1 group (Figure 5C). Elisa analysis indicated that in the M + QC + sh-HIF-1α group, the expression levels of IL-6 and TNF-α were strikingly attenuated when compared to the M + QC + sh-NC group (Figure 5D). There was a similar result when comparing M + QC + sh-HIF-1α + HMGB1 with the M + QC + sh-NC + HMGB1 group (Figure 5D). We further analysis the expression levels of Kim-1, NAGL, IL-6 and TNF-α using western blotting. We found that the expression levels of these factors were significantly reduced in the M + QC + sh-HIF-1α group compared to the M + QC + sh-NC group when compared the M + QC + sh-HIF-1α + HMGB1 group to the M + QC + sh-NC + HMGB1 group, a similar result was found (Figure 5E). These findings indicated that quercetin inhibited the lncRNA NEAT1/HMGB1 signalling pathway via attenuating HIF-1α and improved cell injury and apoptosis in the CI-AKI model.

## Discussion

Accumulating studies have suggested that *Abelmoschus manihot* plays an important role in antioxidant and anti-inflammatory effects. For example, there are studies that report that extract of *Abelmoschus manihot* possesses significant anti-inflammatory activity, and the activity is dose-dependent (Jain and Bari 2010). The antioxidant effect of *Abelmoschus manihot* was also widely studied (Li et al. 2016). Studies also showed that *Abelmoschus manihot* was involved in antioxidant and anti-inflammatory effects via inhibiting the expression of HIF-1α. But there is no report on whether *Abelmoschus manihot* relieves the process of AKI and if so, what are the function and the underlying mechanism.

Quercetin is the main active component in *Abelmoschus manihot*, so, we decided to explore if quercetin also plays a key role in improving the process of AKI. In our present study, we make use of a cell model of CI-AKI to explore the effect of quercetin on cell injury and apoptosis of the CI-AKI model. Moreover, few studies reported that quercetin had an antioxidant effect by regulating the expression of HIF-1α (Roshanzamir and Yazdanparast 2014). In the present study, we showed for the first time that quercetin reduces the inflammatory response of cells in the CI-AKI model via inhibiting HIF-1α, further downregulated the expression levels of lncRNA NEAT1 and HMGB1. Quercetin attenuated the expression of lncRNA NEAT1 by inhibiting HIF-1α and further affected the HMGB1 signalling pathway. Our results showed that the expression of HIF-1α was strikingly increased in the CI-AKI model while silencing of HIF-1α significantly reduced cell injury and apoptosis suggesting that HIF-1α plays an important role in the process of CI-AKI. As one of the important components of *Abelmoschus manihot*, quercetin was reported to attenuate the expression of HIF-1α (Roshanzamir and Yazdanparast 2014). There are no data available regarding whether quercetin slows down the process of CI-AKI via acting on HIF-1α. Our results showed that quercetin at 20 μM significantly attenuated the expression of HIF-1α, and further relieves cell injury and apoptosis in the CI-AKI model.

Evidence has shown that lncRNA NEAT1 plays a crucial role in the inflammatory response process of CI-AKI. Previous high-throughput sequencing results showed that the expression of lncRNA NEAT1 was strikingly upregulated in the rat model of CI-AKI. In addition, some studies also reported that lncRNA NEAT1 improved the expression of HMGB1, further induced cell injury and apoptosis in the CI-AKI model (Zhou et al. 2020). Consistent with previous studies, our results showed that lncRNA NEAT1 and HMGB1 were significantly increased in the CI-AKI cell model. Silencing of lncRNA NEAT1 strikingly suppressed the expression of HMGB1, further relieving the inflammatory response, cell injury and apoptosis. Moreover, our study was the first to report that quercetin restrained the expression of HIF-1α, further attenuated lncRNA NEAT1 and HMGB1 to relieve cell injury and apoptosis in the CI-AKI model.

Despite the crucial findings, some deficiencies also existed in our study. The cell model of CI-AKI used in our study may not reflect the whole process or exact physiological mechanisms of CI-AKI in patients. The effect of factors in the blood may be ignored because the cell culture is *ex vivo*. So, the application of quercetin on this cell model of CI-AKI may not reveal the true effect of quercetin on CI-AKI, for example, quercetin *in vivo* may be partly metabolised in vessels, so the concentration of quercetin between *in vivo* and *ex vivo* may be a little different. More experiments performed in animals or humans are needed to reveal the exact action of quercetin *in vivo*.

Taken together, our results indicate that quercetin relieves cell injury and apoptosis further suppresses the inflammatory response in cell mode of CI-AKI by inhibiting the HIF-1α/lncRNA NEAT2/HMGB1 signalling pathway. Our findings may provide a novel therapeutic target for CI-AKI.

## Data Availability

The datasets used or analysed during the current study are available from the corresponding author on reasonable request.
